# Biochemical Functions of Glutathione S-Transferase Family of *Salix babylonica*

**DOI:** 10.3389/fpls.2020.00364

**Published:** 2020-04-03

**Authors:** Xiang-Lin Zhuge, Hui Xu, Zhi-Jing Xiu, Hai-Ling Yang

**Affiliations:** ^1^College of Biological Sciences and Technology, Beijing Forestry University, Beijing, China; ^2^State Key Laboratory of Systematic and Evolutionary Botany, Institute of Botany, Chinese Academy of Sciences, Beijing, China

**Keywords:** glutathione S-transferase, gene expression, enzyme activity, kinetic analysis, *salix babylonica*

## Abstract

Glutathione S-transferases (GSTs) are ubiquitous enzymes that are encoded by a large gene family, and they contribute to the detoxification of endogenous or xenobiotic compounds and oxidative stress metabolism in plants. Although the GSTs gene family has been reported in many land plants, our knowledge of the evolution and function of the willow GSTs is still limited. In this study, 22 full-length GST genes were cloned from *Salix babylonica* and divided into three classes based on the conserved domain analysis, phylogenetic tree and gene structure: tau, phi and DHAR. The tissue-specific expression patterns were substantially different among the tau and phi GSTs. The *Salix* GST proteins showed functional divergences in the substrate specificities, substrate activities and kinetic characteristics. The site-directed mutagenesis studies revealed that a single amino acid mutation (Ile/Val53→Thr53) resulted in the lowest activity of SbGSTU7 among the *Salix* GSTs. These results suggest that non-synonymous substitution of an amino acid at the putative glutathione-binding site may play an important role in the divergence of enzymatic functions of *Salix* GST family.

## Introduction

Glutathione S-transferases (GSTs; EC 2.5.1.18) are encoded by a large gene family and widely distributed in both prokaryotes and eukaryotes. They are multifunction proteins whose functions include detoxification, since they mainly catalyze the detoxification of a series of xenobiotics by conjugating the reduced glutathione (GSH) to various hydrophobic and electrophilic compounds (Dirr et al., 1994; Armstrong, 1997). GSTs are divided into at least four major families of proteins: cytosolic GSTs, mitochondrial GSTs, microsomal GSTs and bacterial fosfomycin-resistance proteins (Oakley, 2005; Allocati et al., 2009), with the cytosolic GST family being the most abundant of the four groups in plants. Based on the amino acid sequence similarity, gene structure and substrate specificity, the plant GSTs have been divided into eight classes, including phi, tau, theta, zeta, lambda, dehydroascorbate reductase (DHAR), tetrachlorohydroquinone dehalogenase (TCHQD) and γ-subunit of the eukaryotic translation elongation factor 1B (EF1Bg) (Edwards et al., 2000; Oakley, 2005; Lan et al., 2009). In addition, two new classes (hemerythrin GST and iota GST) were recently identified in nonvascular plants (Liu et al., 2013). The GSTs classes of tau and phi are the most abundant in terrestrial plants among these ten GST classes, except in non-vascular plants that have no tau GSTs (Frova, 2006; Liu et al., 2013; Labrou et al., 2015); they were observed only in plants (Basantani and Srivastava, 2007) and are largely responsible for the detoxification processes of endogenous or xenobiotic compounds and oxidative stress metabolism, they also help to select herbicide in crops and weeds (Skipsey et al., 2005; Benekos et al., 2010; Cummins et al., 2011, 2013). In addition to detoxification, GSTs are also capable of regulating the redox homeostasis in cells to protect them against UV radiation and oxidative stress (Loyall et al., 2000; Roxas et al., 2000; Jiang et al., 2010). The other typical functions of GSTs include the involvement in the process of cell signaling and cellular apoptosis and the contribution to the biosynthesis and transport of secondary metabolites (Loyall et al., 2000; Dixon et al., 2010).

The genus *Salix*, a member of the *Salicaceae* family, is widespread throughout China, and represents an essential part of the urban and rural ecosystems (Wu et al., 2015). The species of *Salix* are highly adaptable ones that can grow in various ecological environments (mountains, plains, sand dunes and polar regions), and could be considered as excellent species for the phytoremediation of heavy metals pollution (Yildirim and Kasim, 2016). GSTs are considered to crucially contribute to the stress tolerance and heavy metals resistance, and although this gene family has been reported in many land plants, our knowledge concerning the evolution and function of willow GSTs is limited. With the aim to further understand the GST supergene family in weeping willow (*Salix babylonica*), we studied its structural and functional characterizations in this study. *S. babylonica* is considered to be a promising species for bioenergy production due to the high biomass yields through the short-rotation coppice systems (Brereton et al., 2012; Cunniff et al., 2015). It is widely planted for the construction of shelterbelts, which protect the agricultural land in the oasis of the Gobi Desert (Dickmann and Kuzovkina, 2014). Therefore, it is an important genus from the economic and ecological aspects. In this study, 22 full-length GST genes from *S. babylonica* were cloned and divided into the classes of tau, phi and DHAR. The gene structural features, tissue-specific expression patterns, enzymatic characteristics and site-directed mutagenesis assay of willow GSTs were integrated in this study to provide a comprehensive understanding of the *S. babylonica* GST gene family.

## Materials and Methods

### GST Genes Identification and Nomenclature

In order to identify the GST genes from *S. babylonica*, the transcriptome database (unpublished) of *S. babylonica* was searched using the TBLASTN program with the default algorithm parameters and 81 full-length GST protein sequences of *Populus trichocarpa*. The GST candidates of *S. babylonica* were then looked up in the National Center for Biotechnology Information (NCBI) Conserved Domains Database^1^ to confirm the presence of typical GST N- and C-terminal domains in the protein structures. Next, primers were designed, based on the identified GST gene sequences, to amplify the genomic and coding sequences of each *S. babylonica* GST (Supplementary Table S1), such that the GST genomic sequences and coding sequences were amplified from the *S. babylonica* DNA and cDNA, respectively. The amplified sequences were then cloned into a pEASY-T3 vector (TransGen, Beijing, China), and sequenced in both directions. Finally, the amplified coding sequences of the *S. babylonica* GST genes were mapped to their corresponding genomic sequences to verify the intron/exon structures.

The *S. babylonica GST*s were named according to the system described by Edwards et al. (2000), such that the name of each gene consists of three parts: the source of the organism, the subfamily name and a progressive number. In this study, we used *Sb* to represent *Salix babylonica*, then the subfamily name was denoted by *GST* plus the logogram of each class. For example, *GSTU, GSTF, DHAR*, correspond to the tau, phi, DHAR classes, respectively, and the full phi *GST* genes names are *SbGSTF1, SbGSTF2*, etc.

### Phylogenetic Analysis and Homology Modeling

The GST protein sequences were separated into two distinct parts according to the N-terminal and C-terminal domains. The protein sequences of the full-length, N-terminal domain and the C-terminal domain were, respectively, aligned using the MUSCLE online service. Next, the alignments were manually further adjusted using the BioEdit v7.0.0 software (Alzohairy, 2011), then the pairwise alignments for the sequence identities were analyzed using the Sequence Identity Matrix in the BioEdit software. The Jones, Taylor, and Thornton (JTT) amino acid substitution model was selected by the ModelGenerator program version 0.85. The phylogenetic tree was constructed following a maximum-likelihood procedure using the PhyML software version 3.1 (Guindon et al., 2009). There were 100 bootstrap replicates. The GRX2 protein was used as an outgroup (Oakley, 2005). The structure of the SbGSTU7 gene was built using the GmGSTU10-10 (Protein Data Bank accession number 4CHS) as a template by the SWISS-MODEL software^2^. The simulated structure of the SbGSTU7 gene was then manually processed using the Discovery Studio 4.0 Client software.

### Tissue-Specific Expression Patterns of the *GST* Genes

To extract the total RNA, we sampled the primary leaves, mature leaves, phloem, roots, buds and flowers from three hydroponic *S. babylonica* trees. The primary leaves with a length of 2–3 cm that were newly-expanded and the mature leaves with a length of 10–12 cm were collected from the shoot top and the middle of the shoot, respectively. After the inverse transcription of the RNA using the RNA PCR Kit (AMV) (TaKaRa, Dalian, China), the real-time PCR (qRT-PCR) was performed using the SYBR Green PCR master mix (Tiangen) and Bio-Rad iQ5 Real-Time PCR system (Bio-Rad, United States). Three biological replicates and three technical ones were performed for each qRT-PCR procedure. The *S. babylonica* actin gene (Supplementary Table S2) was used as the internal reference. The relative expression levels were calculated using the 2^–Δ*Ct*^ method (Livak and Schmittgen, 2001), where ΔCT denotes the difference between the target and housekeeping gene actin in the threshold cycles: ΔCT = CT (a target gene) – CT (actin gene). The specific qRT-PCR primers for the *S. babylonica* GSTs are listed in Supplementary Table S2.

### Expression and Purification of the GST Proteins

In this study, 22 GSTs (14 tau, 6 phi, and 2 DHAR) were selected for the enzymatic activity assay. Each of these 22 GST proteins was subcloned into a pET30a expression vector to obtain a 6 × His-tag. The primers that were used to construct the GST expression vectors are listed in Supplementary Table S3. After confirming the sequence of the recombinant vectors, the vectors were introduced into *Escherichia coli* BL21 (DE3) cells. The BL21 cells, containing the recombinant vectors, were then cultured to an optical density (A_600_) of 0.5, and isopropyl-β-D-thiogalactopyranoside (IPTG) was added to the culture to induce the expression of the GST proteins. The final concentration of IPTG was 0.1 mM. After the induction (12 h at 20°C), the cells were harvested by centrifugation (10,000 × *g*, 3 min, 4°C) and resuspended in binding buffer (20 mM sodium phosphate, 0.5 M NaCl and 20 mM imidazole, pH 7.4). The cells were disrupted by sonication in ice-cold binding buffer. Next, the homogenate was centrifuged (10,000 × *g*, 10 min, 4°C). In order to check the solubility of the recombinant proteins, the resulting particulate material and a small portion of the supernatant were analyzed using SDS-PAGE. Regarding the soluble recombinant proteins, the rest of the supernatant was loaded onto a Nickel-Sepharose High Performance column (GE Healthcare BioSciences), and the GST proteins were then eluted with elution buffer (20 mM sodium phosphate, 0.5 M NaCl and 500 mM imidazole, pH 7.4).

In order to obtain the mutated proteins, the site-directed mutagenesis of the protein sequences was performed by the methods that were previously reported (Zeng and Wang, 2005). The primers that were used to construct the mutants are shown in Supplementary Table S3.

### Activity and Kinetics Assays of the *S. babylonica* GST Proteins

The enzymatic activity assay of the purified *S. babylonica* GST proteins were performed using a 752 UV visible single beam spectrophotometer (Jinghua, Shanghai, China). According to the methods described by Habig et al. (1974) and Ricci et al. (1994), we were able to determine the activity of four conventional substrates: 1-chloro-2,4-dinitrobenzene (CDNB), 7-chloro-4-nitrobenzo-2-oxa-1,3-diazole (NBD-Cl), 1,2-dichloro-4-nitrobenzene (DCNB) and 4-nitrobenzyl chloride (NBC). The cumene hydroperoxide (Cum-OOH) was selected to determine the GSH-dependent peroxidase activities, the dehydroascorbic acid (DHA) was selected to measure the dehydroascorbate reductase activities, and the diphenyl ether (fluorodifen) was used as a substrate to determine the herbicide detoxification activities. These activities were determined following the method of Edwards and Dixon (2005), and the reactions were performed in the following buffers: 50 mM potassium phosphate buffer pH 6.5 for CDNB and NBC, 50 mM potassium phosphate buffer pH 7.5 for DCNB, 100 mM sodium acetate buffer pH 5.0 for NBD-Cl, 100 mM potassium phosphate buffer pH 6.5 for DHA, 50 mM glycine pH 9.5 for fluorofen, 50 mM potassium phosphate buffer pH 7.0 containing 1 mM ethylenediamine tetraacetic acid and 1 mM sodium azide for Cum-OOH. The protein concentrations were determined by absorption at 280 nm.

In order to examine the steady-state kinetic parameters of the *Salix* tau GSTs, the apparent *K*_m_ values for GSH and CDNB were separately determined. The concentration of GSH ranged from 0.1 mM to 1 mM, and the concentration of CDNB was fixed at 1.0 mM to determine the *K*${}_{\text{m}}^{\text{GSH}}$. To determine the *K*${}_{\text{m}}^{\text{CDNB}}$ value, the concentration of CDNB ranged from 0.1 mM to 2.0 mM, and the concentration of GSH was fixed at 1.0 mM. The kinetic parameters were calculated using nonlinear regression analysis by the Hyper32 program available online at http://hyper32.software.informer.com/. All the activity and kinetics assays were determined at 25 °C and performed at least three times. The statistical analysis for the enzyme activities between the wild type and mutant proteins were analyzed using the SPSS software ver. 16.0 (IBM Corporation, Armonk, NY, United States).

## Results

### Identification of the *Salix babylonica GST* Genes

**FIGURE 1:**
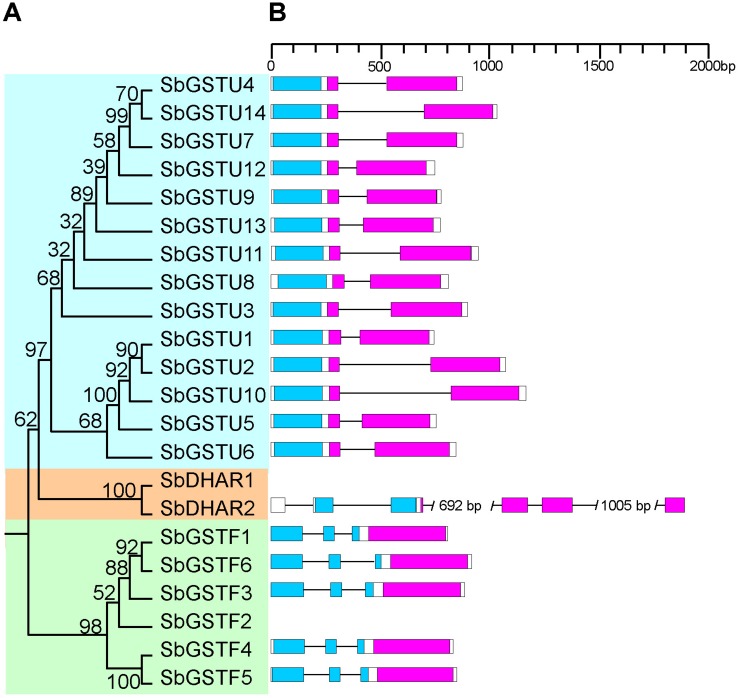
Phylogenetic tree of *Salix* GSTs **(A)** and their gene structures **(B).** The tree was reconstructed with protein sequences using ML procedure with JTT model. Numbers at each node in the ML tree signify bootstrap values. Different GST classes are shaded with different colors. The GST N-terminal domain and C-terminal domain are highlighted in blue and purple, respectively. Introns are indicated as lines.

**FIGURE 2:**
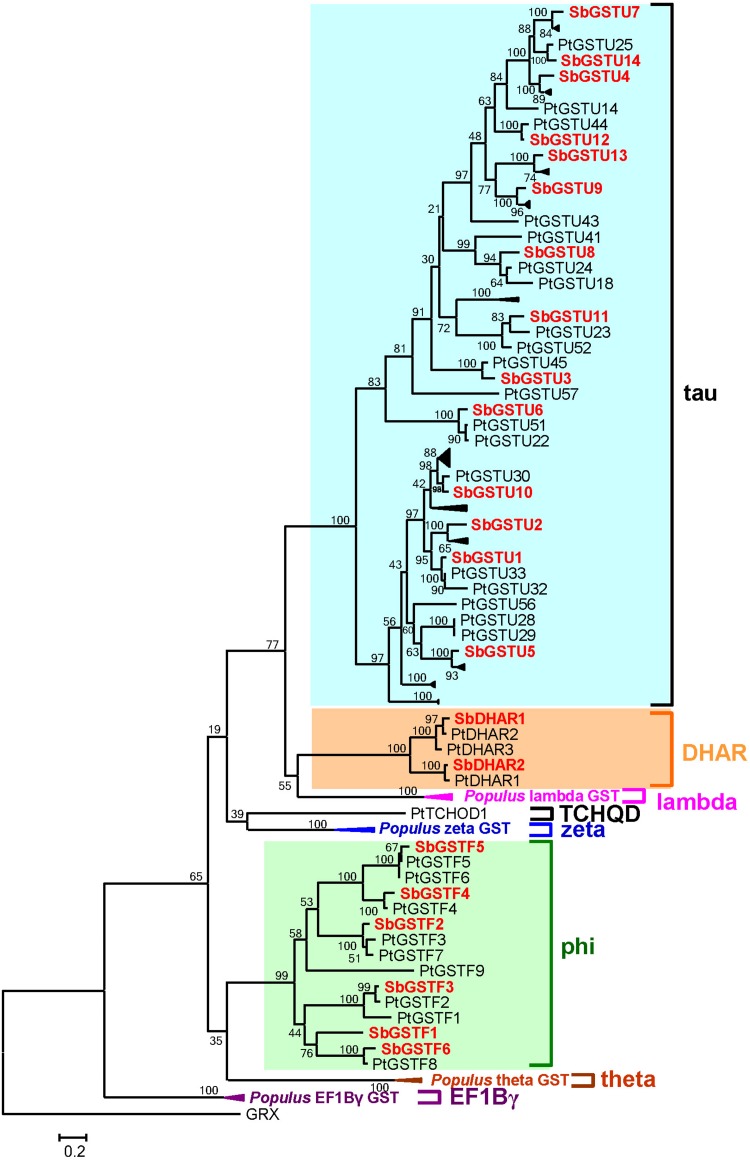
Phylogenetic relationships between *Salix* and *Populus* GSTs. The tree was reconstructed with protein sequences using ML procedure with JTT model. *Salix* GSTs are represented by red letters.

Based on the analysis by the conserved domain of the National Center for Biotechnology Information (NCBI), 22 putative *Salix* GST proteins were identified to belong to the GST classes of tau, phi or DHAR, and their coding sequences were successfully cloned from *Salix babylonica* (Table 1). The phylogenetic relationships revealed that the 22 *Salix* GSTs were divided into three distinct clades with high bootstrap supports (Figure 1A). In order to further confirm the subfamily designations of these GSTs, the phylogenetic tree was constructed using 22 *Salix* and 81 *Populus* GSTs, which indicates that the tau, phi and DHAR GSTs of *Salix* were clustered with the corresponding classes of *Populus* GSTs with high bootstrap support (Figure 2). The results of the conserved domain analysis and the phylogenetic trees showed that among the 22 *Salix* GSTs, 14, 6, and 2 members belonged to the classes of tau, phi and DHAR, respectively.

**TABLE 1:** The GST genes cloned from *Salix babylonica*.

Class	Gene	GenBank accession numbers	Predicted protein molecular weight	Predicted length (amino acids)	Predicted isoelectric point
Tau	*SbGSTU1*	MK300931	25340.33	219	6.64
	*SbGSTU2*	MK300932	25300.47	220	6.36
	*SbGSTU3*	MK300933	25535.78	220	5.77
	*SbGSTU4*	MK300934	24893.03	219	5.50
	*SbGSTU5*	MK300935	25262.11	220	5.07
	*SbGSTU6*	MK300936	25492.7	229	6.85
	*SbGSTU7*	MK300937	24755.59	219	5.28
	*SbGSTU8*	MK300938	26434.38	231	5.19
	*SbGSTU9*	MK300939	25172.49	215	5.93
	*SbGSTU10*	MK300940	25400.54	220	6.20
	*SbGSTU11*	MK300941	26100.15	224	5.83
	*SbGSTU12*	MK300942	25566.54	222	5.09
	*SbGSTU13*	MK300943	25679.82	222	5.83
	*SbGSTU14*	MK300944	25014.03	218	5.28
Phi	*SbGSTF1*	MK300925	24276.81	210	5.91
	*SbGSTF2*	MK300926	24360.02	217	6.38
	*SbGSTF3*	MK300927	24488.79	218	5.99
	*SbGSTF4*	MK300928	23731.45	213	6.50
	*SbGSTF5*	MK300929	23246.65	213	6.75
	*SbGSTF6*	MK300930	24485.17	214	5.24
DHAR	*SbDHAR1*	MK300923	23504.07	212	5.40
	*SbDHAR2*	MK300924	30056.65	270	6.32

### Sequence and Structural Characteristics of *Salix* GSTs

This study identified the gene structures of 20 *Salix* GSTs (Figure 1B), while the gene structure identification of the remaining 2 GSTs, *SbGSTF2*, and *SbDHAR1*, failed due to unsuccessful cloning of their genomic sequences. The gene structures were conserved among the *Salix* GSTs from the tau and phi classes, respectively (Figure 1B). All the 14 tau GST genes contained two exons, and all the six phi GST genes contained three exons. The DHAR GSTs contained more introns than the tau and phi GSTs. The gene of *SbDHAR2* contained five introns which was similar to the poplar DHAR class GSTs. The obtained gene structures in different classes further supported the class designations.

**FIGURE 3:**
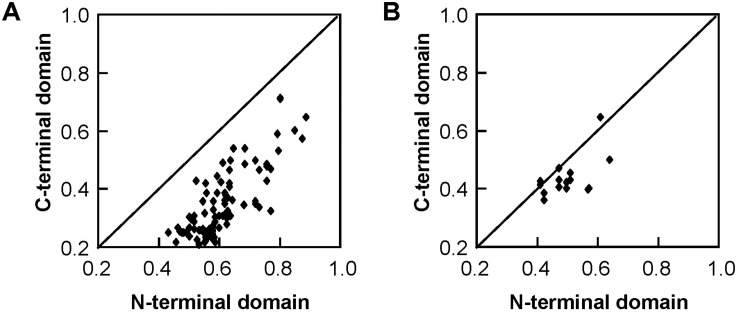
Pairwise protein sequence identity plots for the C-terminal domain versus the N-terminal domain of *Salix* tau **(A)** and phi **(B)** GSTs. Each dot represents the pairwise protein sequence identity for C-terminal domain versus the N-terminal domain between two GST proteins from the same class.

The 22 *Salix* GST proteins’ lengths ranged from 210 to 270 amino acids, and their deduced protein molecular weights were between 23.2 and 30.1 kDa (Table 1). The GST proteins were divided into two distinct domains: The N-terminal domain and the C-terminal domain. The sequence of 14 tau GSTs proteins showed 31.2–75.0% pairwise sequence identities, such that the pairwise protein sequence identity of the N-terminal and C-terminal domains was 43.2–88.4% and 21.0–71.6%, respectively. On the other hand, the pairwise sequence identity of 6 phi GST proteins was 37.3–62.1%, such that it was 41.2–64.1% for their N-terminal domain protein sequences and 36.0–64.2% for the C-terminal domain. Compared with the C-terminal domain sequences, the N-terminal domain sequences were much more conserved for the *Salix* tau and phi GSTs (Figure 3).

### Expression Patterns of *Salix* GSTs

**FIGURE 4:**
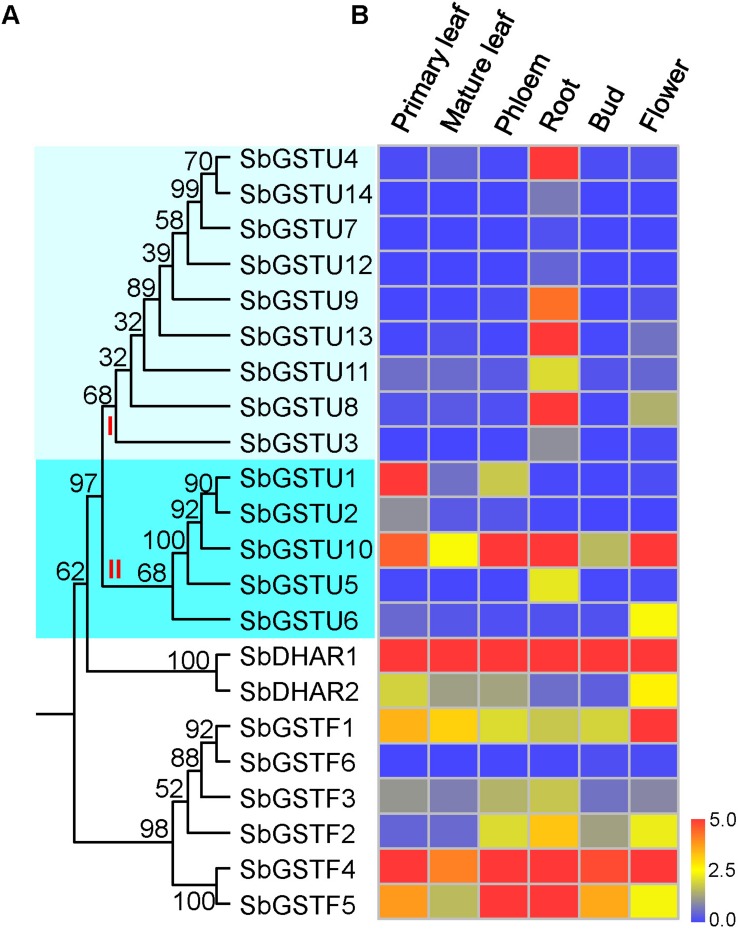
Phylogenetic tree of *Salix* GSTs **(A)** and their expression patterns **(B).** In **(A)**, the phylogenetic tree is from Figure 1. *Salix* tau GSTs are grouped into two clades (clade I and II). The relative expression levels of GST genes by real-time PCR are shown in **(B)**. Color scale indicates normalized expression level [2^(−*Delta*CT)]. The values of relative gene expression level greater than five are represented by the color scale of five. The relative gene expression levels from low to high are represented with color scale from blue to red.

We examined the expression patterns of 22 *Salix* GSTs under normal growth conditions using the quantitative real-time PCR method (Figure 4). The expressions pattern of the *Salix* tau and phi GSTs were different (MRPP test *P*< 0.06), since they were more variable among the *Salix* tau GSTs compared with the phi GSTs. The *Salix* tau GSTs were divided into two clades (Figure 1A). Interestingly, all the nine GST genes in clade I, including the genes of *SbGSTU7* and *SbGSTU12*, showed a much higher expression level in the root tissues than that in the other examined tissues (Figure 4). For the genes in clade II, the genes of *SbGSTU1* and *SbGSTU2* showed a higher expression level in the primary leaf tissues than that in the other examined tissues. The gene of *SbGSTU5* showed a specific high expression level in the root tissues, while the gene of *SbGSTU6* was highly expressed in the flower tissues. The gene of *SbGSTU10* was highly expressed in all the examined tissues (Figure 4).

Regarding the *Salix* phi GSTs, the gene of *SbGSTF1* showed a high expression level in the flower tissues (Figure 4), and the *SbGSTF5* and *SbGSTF3* genes were highly expressed in the phloem and root tissues than the other examined tissues. Among all the six *Salix* phi GSTs, only the *SbGSTF6* gene showed a low expression level in all the examined tissues. Similar to *SbGSTF4*, the gene of *SbDHAR1* was highly expressed in all the tissues. Finally, *SbDHAR2* showed a high expression level in the primary leaf and flower tissues.

### Enzyme Activities of the *Salix* GST Proteins

**FIGURE 5:**
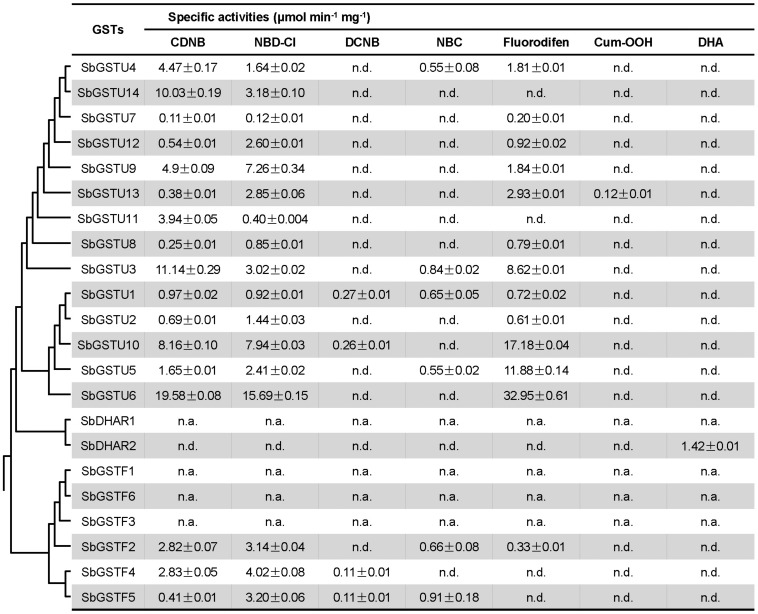
Enzymatic activities of Salix GSTs. The phylogenetic tree is from Figure 1. Values of enzymatic activities to different substrates are means ± SD as calculated from three replicates. n.d., no activity detected; n.a., not assayed.

In order to investigate the substrate specificities and activities of the *Salix* GST proteins, all the 22 *Salix* GST proteins were selected in this study for protein expression and purification. Eighteen *Salix* GSTs (all the 14 tau GSTs, 3 phi GSTs and the SbDHAR2 gene) were expressed as soluble proteins in *Escherichia coli (E. coli)*, while 3 phi GSTs (The genes of SbGSTF1, 3, and 6) and the SbDHAR1 gene were expressed as inclusion bodies in *E. coli.* In order to assay the enzymatic activities of *Salix* GSTs, seven GST substrates were used: CDNB, NBD-Cl, DCNB, NBC, fluorodifen, Cum-OOH, and DHA (Figure 5).

All the 14 *Salix* tau GSTs showed activities to the two substrates of CDNB and NBD-Cl. Among these GSTs, SbGSTU1 showed the widest substrate spectrum with enzymatic activities to five substrates, and SbGSTU6 showed the highest enzymatic activities to the CDNB, NBD-Cl and fluorodifen substrates, to which SbGSTU7 displayed the lowest enzymatic activities. Only SbGSTU13 showed an activity toward the substrate Cum-OOH, and only SbGSTU1 and SbGSTU10 displayed activities toward the substrate DCNB.

All the 3 purified *Salix* phi GSTs showed activities toward the CDNB and NBD-Cl substrates and had no activity toward the Cum-OOH and DHA substrates. SbGSTF4 and SbGSTF5 displayed activities to the DCNB substrate, while SbGSTF2 did not. On the other hand, SbGSTF2 and SbGSTF5 showed activities toward NBC, while SbGSTF4 did not. Only SbGSTF2 exhibited an enzymatic activity toward the fluorodifen substrate.

### Kinetic Properties of the *Salix* GSTs

The *K*_m_ values indicate the affinity of the enzyme and its substrates, and GSTs can catalyze the conjugation of reduced GSH to the electrophilic group of a wide range of hydrophobic compounds. In this study, the steady-state kinetic parameters of 14 purified *Salix* tau GSTs were determined (Table 2). Except for SbGSTU2 and SbGSTU7, the apparent *K*${}_{\text{m}}^{\text{GSH}}$ values of the *Salix* tau GSTs ranged from 0.140 to 0.893 mM, while the range of variations of the apparent *K*${}_{\text{m}}^{\text{CDNB}}$ values was from 0.156 to 5.121 mM, which was greater than the variations of the *K*${}_{\text{m}}^{\text{GSH}}$ values; this suggests that the tau GSTs had similar, high affinities for the GSH substrate than the hydrophobic substrates. Among the 14 *Salix* tau GSTs, SbGSTU6 showed the highest *k*${}_{\text{cat}}^{\text{CDNB}}$ value, SbGSTU10 had the highest (*k*_cat_*/K*_m_)^CDNB^ value, and SbGSTU7 had the lowest *k*${}_{\text{cat}}^{\text{CDNB}}$ and (*k*_cat_*/K*_m_)^CDNB^ values.

**TABLE 2:** Steady-state kinetic parameters of *Salix* tau GSTs.

GSTs	*K*${}_{\text{m}}^{\text{GSH}}$	*k*${}_{\text{cat}}^{\text{GSH}}$	(*k*_cat_*/K*_m_)^GSH^	*K*${}_{\text{m}}^{\text{CDNB}}$	*k*${}_{\text{cat}}^{\text{CDNB}}$	(*k*_cat_*/K*_m_)^CDNB^
	mM	s^–1^	mM^–1^s^–1^	mM	s^–1^	mM^–1^s^–1^
SbGSTU1	0.473 ± 0.023	6.283 ± 0.149	13.277	0.201 ± 0.004	6.056 ± 0.081	30.102
SbGSTU2	0.996 ± 0.007	2.496 ± 0.046	2.506	4.819 ± 0.273	6.383 ± 0.122	1.325
SbGSTU3	0.140 ± 0.004	16.714 ± 0.127	119.300	1.003 ± 0.033	28.819 ± 0.783	28.733
SbGSTU4	0.146 ± 0.002	2.963 ± 0.025	20.296	5.121 ± 0.106	24.482 ± 0.371	4.781
SbGSTU5	0.240 ± 0.011	3.376 ± 0.011	14.073	2.364 ± 0.080	7.782 ± 0.071	3.292
SbGSTU6	0.141 ± 0.002	16.483 ± 0.131	116.572	2.266 ± 0.048	112.987 ± 2.687	49.862
SbGSTU7	1.183 ± 0.059	0.804 ± 0.025	0.679	2.074 ± 0.165	1.415 ± 0.015	0.682
SbGSTU8	0.893 ± 0.026	1.197 ± 0.021	1.340	2.628 ± 0.669	2.061 ± 0.148	0.784
SbGSTU9	0.208 ± 0.006	7.564 ± 0.089	36.365	2.785 ± 0.060	21.060 ± 0.391	7.562
SbGSTU10	0.284 ± 0.009	7.391 ± 0.028	26.053	0.156 ± 0.002	16.183 ± 0.178	104.004
SbGSTU11	0.732 ± 0.082	16.302 ± 0.326	22.285	3.372 ± 0.538	22.472 ± 2.083	6.664
SbGSTU12	0.333 ± 0.054	1.314 ± 0.013	3.947	2.144 ± 0.272	1.934 ± 0.043	0.902
SbGSTU13	0.262 ± 0.002	11.400 ± 0.092	43.529	1.273 ± 0.062	14.948 ± 0.436	11.742
SbGSTU14	0.491 ± 0.029	12.281 ± 0.132	25.022	4.269 ± 0.396	41.823 ± 1.633	9.797

*Values shown are means ± SD, calculated from three replicates.*

### Site-Directed Mutagenesis of the SbGSTU6 and SbGSTU7 Proteins

**FIGURE 6:**
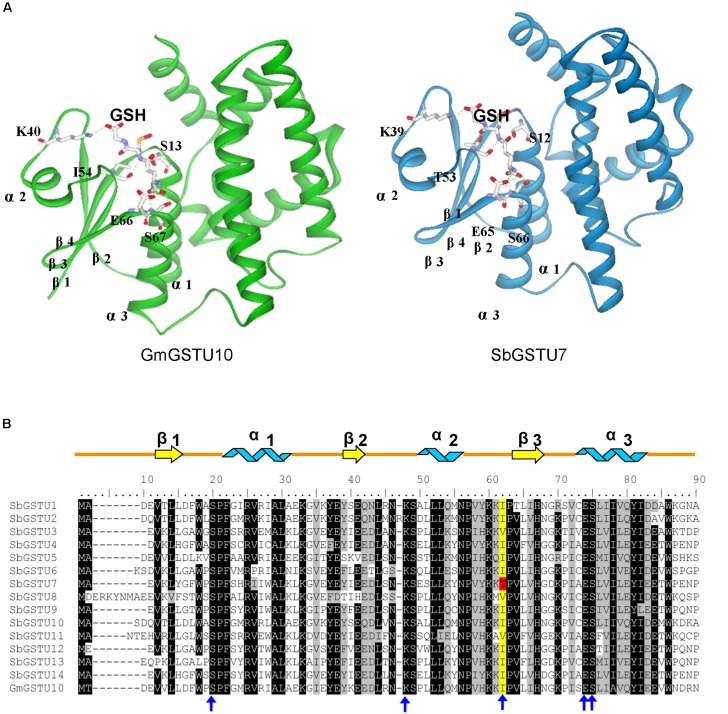
Structural comparison of G-site residues **(A)** and N-terminal amino acid sequence alignment of the *Salix* tau GSTs **(B).** The structure of GmGSTU10 (template, PDB No. 4CHS) and SbGSTU7 (stimulated) are presented in **(A)**. The amino acid structures of GSH-binding sites are shown with sticks. In **(B)**, the conserved amino acids are shaded with black and gray. The G-sites of SbGSTU7 are marked with blue arrows. One conserved residue of G-sites (Ile/Val, alignment position 62) is shaded with yellow and the Thr substitution in SbGSTU7 of this residue is shaded with red. Alpha helices and beta strands are represented as blue ribbons and yellow arrows, respectively.

**FIGURE 7:**
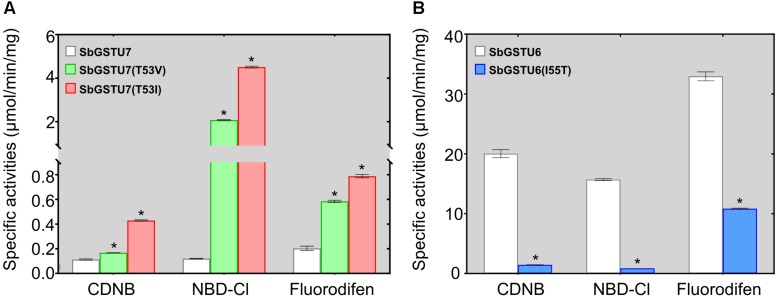
Enzymatic activities of the SbGSTU7 mutants **(A)** and SbGSTU6 mutant **(B)**. Data for each sample represent the average of three biological replicates, and bars indicate the standard deviation; * significant difference (one tail *t*-test, *P* < 0.01) in enzymatic activities between the wild-type and its mutants.

Among the 14 *Salix* tau GSTs, SbGSTU7 showed the lowest enzymatic activities to the CDNB, NBD-Cl and fluorodifen substrates, to which SbGSTU6 had the highest enzymatic activities (Figure 5). The predicted tertiary structure of SbGSTU7 was similar to that of the *Glycine max* tau GST (GmGSTU10, Protein Data Bank code (ID) No.: 4CHS). The GSH-binding sites of GmGSTU10 were Ser13, Lys40, Ile54, Glu66, and Ser67 (Figure 6A). Based on the N-terminal amino acid sequence identity, these sites were highly conserved among the 14 *Salix* tau GSTs (Figure 6B). However, the conserved Ile/Val residue (Corresponding to the Ile54 site of GmGSTU10) is substituted by Thr in SbGSTU7 (Figure 6B). In order to verify whether this substitution was responsible for the low enzymatic activities of the SbGSTU7 protein, two groups of mutants were constructed. Firstly, the Thr53 residue of SbGSTU7 was, respectively, mutated to Ile and Val residues. Compared with SbGSTU7, the enzymatic activities of the mutants T53V and T53I to the CDNB, NBD-Cl and fluorodifen substrates were much higher (Figure 7A). Secondly, this study mutated the Ile55 residue of SbGSTU6 to a Thr residue. Compared with SbGSTU6, the mutant I55T showed much lower enzymatic activities to the CDNB, NBD-Cl and fluorodifen substrates (Figure 7B).

## Discussion

Twenty-two full-length GST genes were cloned from *Salix babylonica* genome. These 22 *Salix* GSTs were divided into tau, phi and DHAR classes. The previous study identified 81 GST genes from the *Populus trichocarpa* genome (Lan et al., 2009), which were divided into eight classes, including the theta, zeta, phi, tau, lambda, DHAR, TCQD, and EF1Bγ GST classes. Compared with the other class GSTs, the tau GST class contained the highest number of members. Similar to *Populus trichocarpa*, the *Salix* tau GSTs contained the highest number of members among these *Salix* GSTs. The gene structures of the tau and phi GSTs in *Salix* were conserved with their homologs in *Populus* (Lan et al., 2009), and their enzymatic characteristics were also similar to those of *Populus*. For example, the *Salix* tau and phi GSTs had a broad substrate spectrum and differentiated substrate activities. In addition, both *Salix* and *Populus* DHAR showed high activity toward the DHA substrate, which distinctly diverged in the enzyme specificity from the other GST classes.

Regarding the GST proteins, there are two distinct domains: The N-terminal and the C-terminal. The N-terminal domain contains α-helices and β-strands, which are arranged in a thioredoxin-like fold. The C-terminal domain is all α-helical. The two domains are connected by a short linker sequence. This study confirmed that the sequences similarities between the N-terminal domain sequences of *Salix* tau and phi GSTs were higher than those of the C-terminal domain sequences (Figure 3), which meant that the N-terminal domain sequences were much more conserved. Similar results were previously observed in other plant GSTs (Lan et al., 2009; Yang et al., 2014; Liu et al., 2015). GSTs catalyze the conjugation of the glutathione thiolate anion with a multitude of second substrates or as non-covalent binding proteins for a range of hydrophobic ligands (Frova, 2003). Besides, the N-terminal domain of GSTs contains a glutathione binding site (G-site) for the common GST substrate (Dirr et al., 1994). These characteristics resulted in highly conserved N-terminal domain structures among different GSTs. On the other hand, the C-terminal domain contains a hydrophobic substrate binding site (H-site) (Dirr et al., 1994), and it could accommodate a diverse range of hydrophobic compounds (Edwards et al., 2000). The diversity of substrates correlates to variable C-terminal domain structures.

In this study, we determined the enzymatic activity of the *Salix* GST proteins. Interestingly, only theGSTU13 protein of *Salix* showed GSH peroxidase activity whereas many GSTs of other species usually presented this activity (Liu et al., 2015; Han et al., 2018). It is possible that the other GSTs of *Salix* could also have GSH peroxidase activity but they are too low to be detected under our measurement conditions or they have GSH peroxidase activity toward different substrates other than cumene hydroperoxide but are not detected in this study. Among the 14 *Salix* tau GSTs, SbGSTU7 showed the lowest enzymatic activities to the CDNB, NBD-Cl and fluorodifen substrates. The Thr53 of SbGSTU7 was a G-site residue, while this residue was Ile/Val in all the other *Salix* tau GSTs. Compared with SbGSTU7, the enzymatic activities of the T53V and T53I mutants to the CDNB, NBD-Cl and fluorodifen substrate were much higher (Figure 7A). These results indicated that the Ile/Val→Thr substitution could result in the low enzymatic activities of SbGSTU7. Thr53 is located in the loop that connects the α-helix 2 to the β-strand 3 in the N-terminal domain (Figure 6A) and plays important roles in the recognition and orientation of GSH (Dirr et al., 1994). Pro54, a neighboring residue of Thr53, is important for the proper folding and packing of the G-site substructures (Zeng et al., 2005), and it was highly conserved in all the *Salix* tau GSTs (Figure 6B). The Ile/Val→Thr substitution might alter the conformation of Pro54 and the loop structure connecting the α-helix 2 to the β-strand 3, and these changes might affect the conformation of the G site in SbGSTU7.

## Conclusion

Twenty-two full-length GST genes were cloned from *Salix babylonica* genome. The *Salix* tau and phi GST proteins showed substantially different tissue-specific expression patterns. In addition, the GST proteins exhibited significant variations in the enzymatic activity. The Ile/Val→Thr substitution resulted in the lowest activity of SbGSTU7 among the *Salix* GSTs. This study suggest that an amino acid at the putative glutathione-binding site may play an important role in the divergence of enzymatic functions of *Salix* GST family.

## Data Availability Statement

The sequence data of GSTs identified in this study was deposited in the National Center for Biotechnology Information (NCBI) under the accession numbers listed in Table 1.

## Author Contributions

H-LY and X-LZ conceived the project. X-LZ and Z-JX performed research. All authors contributed to data analysis, writing of the manuscript, and reviewed the manuscript.

## Conflict of Interest

The authors declare that the research was conducted in the absence of any commercial or financial relationships that could be construed as a potential conflict of interest.

## Footnotes

1 http://www.ncbi.nlm.nih.gov/Structure/cdd/wrpsb.cgi

2 https://swissmodel.expasy.org/interactive

## Supplementary Material

TABLE S1 Primers used to amplify *Salix babylonica* GST genes.

TABLE S2 PCR primers used to detect the expression of *Salix babylonica* GST genes.

TABLE S3 Primers used to construct the protein expression vector.
